# Exploring the Lived Experience of Missed Nursing Care in Postgraduate Nursing Students in Iran

**DOI:** 10.30476/ijcbnm.2020.85865.1344

**Published:** 2021-01

**Authors:** Fatemeh Najafi, Alireza Nikbakht Nasrabadi, Leila Mardanian Dehkordi

**Affiliations:** 1 Department of Medical Surgical Nursing, School of Nursing and Midwifery, Tehran University of Medical Sciences, Tehran, Iran; 2 Department of Adults Health Nursing, School of Nursing and Midwifery, Isfahan University of Medical Sciences, Isfahan, Iran

**Keywords:** Experiences, Missed care, Nursing students, Phenomenology

## Abstract

**Background:**

Missed care is a global phenomenon, which can include many clinical conditions that threaten the patients’ safety in all countries and cultures, and also indicates the quality of nursing care. The nursing students’ awareness and understanding of missed nursing care is of great importance. The current study aims to explore the lived experience of postgraduate nursing students in missed care.

**Methods:**

The current qualitative study was performed based on the interpretive phenomenological approach in Tehran, Iran, in February to December 2019. A total of 10 master’s degree nursing students were selected through purposive sampling. A total of 10 semi-structured individual interviews were used to collect the data. The trail version of MAXQDA-10 software was used for coding. All interviews were recorded and codified, and the main themes were extracted from them using Dicklemann et al.’s (1989) analytical method.

**Results:**

Two main themes, five sub-themes, and 31 meaning units were obtained. The main themes included: “unfulfilled care” and “living in limbo”.

**Conclusion:**

Missed care, as unfulfilled care, is accompanied with living in limbo for nursing students, and this condition is influenced by organizational and personal factors. It seems that managers can prevent missed nursing care by supervising nursing care, reducing the nurses’ workload, creating a sense of commitment to work, and enforcing ethical issues among nurses.

## INTRODUCTION

Nurses work in complex environments where technology and practice are constantly changing; hence, they need more competencies to provide quality care to their clients. ^1 , 2^ Patient care depends on the treatment and involves nurturing, professional activities, processes, and crucial decision-making. In other words, patient care is the spiritual, physical, and mental provision of nursing services to clients and their families. ^3^ Clients expect all care and treatment services to be delivered on time without any delay and with adequate skills, accuracy, and continuity. ^4^ They want to be cared for by knowledgeable, trustworthy, and communicative people with mutual understanding. ^5^

Missed nursing care refers to any aspect of missed or postponed care, and is described as an unintentional error. ^6 - 8^ The health care professionals, especially nurses, play an important role in providing safe care to patients. Nursing practice environment is recognized as an important predictor of the quality of care and its consequences for the patient. ^9^ Missed nursing care reflects the quality of nursing care, ^7 , 8^ and one of the most important components of the quality of care is patient safety. ^10^ Patient safety is affected by errors of commission (such as wrong side marking in eye surgery) and errors of omission (such as not ambulating the patient). ^11^ The errors of commission were evaluated in many studies, while errors of omission have not received much attention. ^11 - 13^ Missed nursing care is a global phenomenon and refers to many clinical conditions that threaten the patient’s safety in all countries and cultures. ^7 , 8^

The prevalence of missed care among Intensive Care Units (ICU) nursing staff is reported 55%-98% worldwide. ^14^ Nine areas of mobility, repositioning patients, delayed or missed feeding, patient education, discharge planning, emotional support, commitment to good hygiene, intake and output documentation, and supervision have been reported as common areas of missed nursing care. ^6 , 7^ Reasons for missed care are often related to nurses, inefficiency in doing tasks, “it’s not my job” syndrome, habit, prolonged involvement with the patient, and denial that are traditionally associated with appropriate nursing care, but it is usually ignored or left incomplete and nurses fail to follow up their duties and use denial as a mechanism to deal with missed care. ^6^ The lack of time management skills; failure to assign the nurses to different wards based on their scientific, physical and mental abilities; and the lack of familiarity with the equipment are mentioned as other reasons for missed nursing care. ^12^

Missed care is consistently associated with negative consequences for the nurse, patient, and organization. ^14^ Studies have reported many negative outcomes related to missed nursing care such as decreased job satisfaction, absenteeism and quitting job. ^15^ Some of the complications of missed care for the patient include falls, medication errors, nosocomial infections, pressure sores, gastrointestinal bleeding, increased pain and discomfort, and readmissions. ^8 , 16 - 18^ Patient transfer to (ICU), cardiac arrest, and death are other catastrophic consequences of missed care. ^18 , 19^ A study referred to prioritization, teamwork, intentional and thoughtful action, and reflection on how these activities are carried out as the strategies of overcoming the barriers to appropriate nursing care. ^8^ Despite the long history of missed care, it has recently been a concern for the nursing profession; also, despite the prevalence of missed care and its importance, few studies have been conducted in Iran on this issue, ^12^ so little is known about its nature and associated factors.

Missed nursing care continues, and this demonstrates the need to address this phenomenon. Since missed nursing care has always been reported alongside medical errors, it is important to have a clear understanding of what health professionals call a threat to the quality of care. Adequate awareness and understanding of the dimensions and issues relevant to this problem is one of the essentials for any decision making, planning, and taking measures by all health decision makers and policymakers. Previous studies on missed care in Iran have been conducted among nurses in clinical setting, ^12 , 20^ but among postgraduate nursing students with clinical and educational experiences, no study has been done to discover the meanings of missed nursing care. Since postgraduate nursing students will have more managerial roles and are more likely to continue their education in the future, selecting this group as the target of this study will have more potential effects for improvement of health care in future. Therefore, a deeper understanding of the unknown aspects of this issue can facilitate the planning of a comprehensive nursing care program and improvement of quality of nursing care. Full understanding of postgraduate nursing students’ experiences is not possible without qualitative research. The researchers in the present study aimed to use interpretive phenomenology because its goal is to better understand the nursing students’ experience of missed nursing care. The purpose of phenomenological research is to describe specific phenomena as lived experience. Phenomenology is the lived experience of human beings from the world of their everyday lives. In fact, it is the experience of a person that tells him what is real and true in his life. These lived experiences give meaning to a person’s perception of a particular phenomenon, which is influenced by all internal and external factors. ^21^ Interpretive phenomenology focuses on the experience of human beings and their interpretations of those experiences. ^22 , 23^ The current phenomenological study aimed to explore the lived experience of postgraduate nursing students regarding missed nursing care and to understand this complex phenomenon and its various dimensions in order to find a way to develop strategies for managing and reducing it.

## METHODS

The present interpretive phenomenology study was conducted to explore and analyze the experience of postgraduate nursing students about missed nursing care in February to December 2019. The study setting was one of the Iranian universities. Data were collected and analyzed simultaneously, after completing the research plan and receiving confirmation and approval by Research Committees of the School of Nursing and Midwifery. The participants were selected from master’s degree nursing students with acceptable clinical practice experiences. These students had passed their internship program and were employed as clinical nurses in teaching hospitals. Invitation letters containing a description of the study objectives were presented to the students, and those who were willing to participate were asked to sign informed consent and plan a specific time for the interviews. Participants were selected using purposive sampling. A total of 10 students including six females and four males were selected and individual, in-depth, face to face semi-structured interviews were then held with them. In order to reach the maximum variation, we considered different conditions in terms of age, gender, specialty, and work experience in different clinical wards. Inclusion criteria were students with at least 1 year of clinical practice experience and study in postgraduate level. Exclusion criteria included unwillingness to continue with the study and withdrawing from the study. 

The number of sessions and the length of each interview varied based on each participant’s condition and depended on factors such as the time and willingness of individuals. The mean time of interviews varied from 30 to 40 minutes and each participant was interviewed once. Before starting the interviews, an initial interview guide was provided and helped the researcher to ask further questions in the field. In the interviews, first a basic and follow-up question such as “What comes to your mind when I say missed care?, What is missed care like?”, “How do you feel when missed care occurs?”, and “Can you give an example”, were asked followed by the in-depth exploratory questions such as: “Can you explain more or When you say ... what do you mean and how ...?, Have you had any similar experiences?, and What do you mean by saying…?” At the end, the participants were asked to comment if there was anything left to say. Interviews were digitally recorded with the permission of the participants and then transcribed verbatim and converted into Microsoft word. The MAXQDA-10 software was used to better manage the data. Interviews continued until we reached the depth, richness, abstraction, and relevance. Data analysis and collection were performed simultaneously; for instance, data analysis was started with the first interview. Finally, by obtaining eligible participants, the data were collected and analyzed simultaneously and saturated within 10 months. The data were analyzed using the Dicklemann et al.’s (1989) approach, a seven-step process based on the Heidegger phenomenology, with a team approach. This step-by-step process was carried out as follows: Each interview was first transcribed verbatim and reviewed one or more times to get a general understanding of the context. For each transcript, a commentary was written by the author to understand and extract the hidden meanings through coding. Team members exchanged their ideas on extracting the topics and themes, and as the interviews continued, previous topics became clearer and evolved, and sometimes new ones emerged. This was done through discussion with team members. By finding new topics, patterns and interpretations were formed and explicit and implicit meanings were extracted from the interview texts. These meanings were not simply the participants’ statements, but also included the interview space and the way the participants answered the questions. In order to clarify, categorize, and eliminate the contradictions in the interpretation, we repeatedly returned to the texts. The purpose of data analysis was to extract the concepts and then the themes. The themes were a set of general characteristics that represent the central meaning of the concepts, similarities, and differences. At each stage, as the work progressed, by the combination of interpretive abstracts, the combined analysis was performed to relate the themes in the best manner. In order to achieve rigor of the study, we considered and used the criteria proposed by Lincoln and Guba in 1985, which include credibility, dependability, confirmability and transferability. ^24^ Therefore, by selecting the appropriate context, resources, and eligible participants; close prolonged and continuous interaction with the participants; adopting a team approach using collective team discussion; doing analytical comparisons and reciprocal referral to raw data, the validity of the study was ensured. 

Ethical consideration was observed through explaining the aims and methods used in the research for the participants, obtaining informed written consent from the research participants, assuring the participants about the confidentiality of their information, explaining the purpose of using the audio recording, reminding the voluntary participation in research, and the possibility of withdrawal at any stage of research. This study was approved by the local research ethics committee of Tehran University of Medical Sciences (IR.TUMS.VCR.REC.1397.727).

## RESULTS

Study participants included six women and four men, with a mean age of 30.6 years, ranging from 23 to 39 years. Four of the participants were married and the rest were single. Also seven of them were in the second semester and three in the fourth semester of the master’s degree program. Eight participants were studying medical-surgical nursing and two were studying geriatric nursing. All participants were working; of whom six had work experience of less than 10 years and four over 10 years. Five participants worked in the (ICU), two in the emergency department, and three in the Cardiac Care Units (CCU). The participants’ demographic information is listed in Table 1.

**Table 1:** Demographic characteristics of the participants

Participant	Age	Sex	Academic field	Semester	work experience	Unit
1	23	Male	Medical-Surgical	Second	1 year and two months	CCU^a^
2	33	Female	Medical-Surgical	Fourth	11 years	Emergency
3	27	Male	Geriatric	Second	5 years	ICU^b^
4	24	Male	Medical-Surgical	Second	2 years	Emergency
5	37	Female	Medical-Surgical	Second	12 years	ICU
6	39	Female	Medical-Surgical	Second	14 years	ICU
7	34	Female	Medical-Surgical	Second	11 years	ICU
8	25	Male	Geriatric	Second	2 years	CCU
9	33	Female	Medical-Surgical	Fourth	6 years	CCU
10	31	Female	Medical-Surgical	Fourth	7 years	ICU

Two main themes emerged in the data analysis process: “Unfulfilled care” and “Living in limbo”.

a Coronary Care Unit;

b Intensive Care Unit

### 1. Unfulfilled Care

This theme represents the majority of the nursing students’ experience of missed care as a missed opportunity that may even lead to death and catastrophic consequences for the patient. It was also considered as a routine care by the participants. Unfulfilled care had three sub-themes of deterioration of opportunity, induction of death, and worthless conception of care, which are explained as follows by the participants’ quotes. The information about the main themes, sub-themes and meaning units is given in Table 2.

**Table 2:** Table of the meaning units, sub-themes and main themes

Meaning units	Subtheme	Theme
Delay in care	Deterioration of opportunity	Unfulfilled care
An irreparable situation		
A Necessity Missed		
Missing circle in providing care		
Unsuccessful care		
Painful outcome	Induction of death	
Equivalent to death		
Pushing to death		
Irreparable outcome		
Negative wave		
Ineffective care	Worthless conception of care	
Inevitable event		
Normalization		
Unnecessary care		
Impatience in providing care		
Trampling on the patient’s right	Ethical conflict	Living in limbo
Discredit		
Discrimination in care		
Being questioned		
Immoral care		
Feeling worthless and inadequate		
Deception and denial		
Ignoring error		
Lack of adequate information and proper training		
Feeling of Regret	The turbulence of existence	
Mental struggle		
Emotional struggle		
Stressful feeling		
Impact on the nurse’s mind		
Concealment of truth		
Disappointment		

1.a. Deterioration of opportunity: 

This sub-theme includes five common meanings, including: delay in care, an irreparable situation, a necessity missed, missing circle in providing care and unsuccessful care. Regarding the meaning of missed care, the participants stated that the patients needed some care, but due to working conditions, it was ignored and a valuable care was missed, and also that care was not completed and cannot be compensated.

Some of the participants’ statements that reflect the deterioration of opportunity are as follows:

*“You find a patient who had received cares that were not delivered on time, so he missed something valuable. For example, you had something so valuable, but you lost it”* (Participant 10). 

*“The patient needed some care, but we were unable to actually provide him with that, because of working conditions, a missed circle in our care, which means no attention is paid to that...”* (Participant 3)

1.b. Induction of death:

This sub-theme includes five common concepts: painful outcome, equivalent to death, pushing to death, irreparable outcome, and negative wave. The participants perceived missed care as an early induction of a critically ill patient’s death, worsening of the patient’s condition, pushing him or her into a bad condition, prolonged treatment, and a catastrophe, which will cause many complications for the patient, nurses and organization. The statements of many of the participants about missed care indicated that this phenomenon is associated with negative feeling, leads to death, and even is equivalent to death, as follows:

*“It is like after death you get the cure; this is the best example I can give. Because what should be done was not done ...”* (Participant 1)

*“The treatment is longer, so it has more complications for the patient. Some of the care that is missed has consequences. They are caused by the same care that is missed and some care that are not delivered, and the consequences can be very severe.”* (Participant 6)

1.c. Worthless conception of care:

This sub-theme includes five common concepts: ineffective care, inevitable event, normalization, unnecessary care and impatience in providing care. Some participants found some cares unnecessary and useless for patients based on their diagnosis and opinions or workplace conditions such as heavy workload or nurses’ fatigue. At their discretion, they prioritized the care that had led to the missing of some important care. Some also argued that documenting is superior to nursing care itself. *“I didn’t change the dressing that I should have done because I was tired or late, or I felt that it shouldn’t be changed because I thought the wound was clean and good ... For example, something like dressing change (e.g. PRN) probably does not hurt the patient. If I’m tired or busy, the patients are ill, or for whatever reason I can’t do it, well, I don’t ..”* (Participant 1.)

Perceiving some care, which were worthless and repetitive such as patient education, was also extracted from some participants’ statements:

*“Sometimes, I felt that the words and trainings were even repeated for the patient and he has heard them before. As a nurse, I thought that this training doesn’t help my patient, it was just something routine, something that he should know, and the care that you consider to be trivial may be missed...”* (Participant 2)

### 2. Living in Limbo 

This theme reflects the ethical conflicts and challenges that may arise in relation to the patient, and the confusions and emotions experienced by nursing students during missed care as expressed in the interviews with various explanations.

2.a. Ethical conflict:

This sub-theme includes nine common concepts: trampling on the patient’s right, discredit, discrimination in care, being questioned, immoral care, feeling worthless and inadequate, deception and denial, ignoring error, lack of adequate information, and proper training. Some nurses do not report missed care to their coworkers for fear of being reprimanded during their shift. They may also think that no one may notice it, but it can lead to loss of patient rights and patient harm. The statements of some participants about the missed care are as follows:

*“Now delivering a service to the patient mistakenly, but not mentioning in to him, or documentation error, whatever intentionally, mistakenly, or due to fatigue, is very bad because it will postpone everything unintentionally...”* (Participant 9)

*“I think being afraid of reprimand, for example, telling a nurse why you didn’t do that, causes the nurse not to report the missed care ...”* (Participant 7)

Some participants also experienced the lack of adequate information and proper training in creating ethical conflicts and missed care:

*“When I don’t have adequate information, it will definitely hurt a patient; different things may happen...”* (Participant 7)

2.b. The turbulence of existence: 

This sub-theme includes seven common concepts: feeling of regret, mental struggle, emotional struggle, stressful feeling, impact on the nurse’s mind, concealment of truth and disappointment. All nursing students also talked about the impact of missed care on nurses. They were so upset while talking about it and their sadness was evident on their faces. When they experience missed care, they experience feelings of regret, remorse, and constant and endless mental distress, which accompany them even long after the shift is over. Some of them have even considered leaving their jobs out of frustration. Inefficient system and insufficient supervision were the factors that caused frustration and negative feelings due to the lost care in nurses. 

A participant said:

*“Your patient is hospitalized and you are at home, while your timing was lost; there would be a feeling inside you that is conscience-stricken or an internal discomfort about which you might not be able to talk to others, but it is inside you; it bothers you and maybe remains in your mind for 2-3 days until you forget ... Such things may affect feeling and job confidence of nurses...”* (Participant 2) 

*“It doesn’t feel good and I’m tormented by my conscience. I’m upset, so I don’t fix it. I say nothing should be missed until the last minute ...”* (Participant 6)

## DISCUSSION

In the current study, several meanings of missed care were extracted from the statements of postgraduate nursing students. Two main themes and five subthemes resulted from data analysis. The main themes included unfulfilled care and living in limbo. 

In a similar qualitative content analysis study in Iranian nurses, similar findings such as lack of motivation of the staff to do interventions that they think were not necessary for the patient as well as ineffective care and a waste of time were reported as components of missed nursing care. Some participants in both studies have stated that sometimes missed nursing care is unavoidable due to systemic and managerial problems. ^12^

In line with a similar study conducted in Norway, emotional stress that emerged as a subtheme in this study explained the nature of missed nursing care. Other findings reported factors such as work constraints, organizational constraints, professional constraints in relation to missed nursing care similar to the present study. ^6^

Unfulfilled care reflects deterioration of opportunity, induction of death, and worthless conception of care that the participants in this study experienced. 

The participants in this study described missed nursing care as an irreparable situation like a missing circle in the care process. In one similar study, missed nursing care was defined as the omission or delay in any part of nursing care required by the patient, which is consistent with some characteristics of missed nursing care in this study. ^25^ According to another study, the basis of missed nursing care is the nurse’s involvement between the routine methods and real empathy for the patient’s recovery, ^26^ which is similar to the meaning of the missing circle in the care process in the present study.

As in the present study, induction of death was interpreted as a painful outcome, equivalent to death, an irreparable outcome, and negative wave of nursing students’ experiences of missed care. Various studies have also mentioned the catastrophic consequences of missed care. In fact, the consequences of missed care include a threat to patient’s safety. ^12^ The results of a study that evaluated why and how nursing care is missed indicated that a large proportion of hospitalized patients are at risk of missed care or nursing errors. ^27^ According to the findings of another study, missed care is associated with negative patient outcomes such as falls, medication errors, hospital infections, increased hospital costs, and death. ^28^ However, compared to other studies, the issue of death, non-existence and irreparability has been more prominent, which may be due to the cultural considerations.

As the findings of the present study indicated, missed care is sometimes caused by the notion that something normal, unnecessary care and impatience in care is missed; the point that was also referred to in the literature on missed nursing care. Review of the literature in this field shows that missed care is a global problem that is concealed by different experiences and different natures. ^14 , 29 , 30^ In this regard, the results of a study showed that nursing staff was used to making no attempt to do some aspects of care. According to the participants in this study, when some aspect of care such as patient reposition is missed, it is easier to set it aside for tomorrow and the following days. If tasks are ignored for a long time, expectations to complete a care disappear. ^25^

In one study, the participants noted that they sought patient satisfaction, but they had lost the art of patient care to paperwork and they were discouraged from neglecting care and paying more attention to paperwork tasks. In the same study, according to the participants, nurses cannot fully care for each patient due to a heavy workload, so the probability of missing nursing care is more and inevitable. ^26^ In another study, perfunctory was identified as the most important underlying cause of missed nursing care. Many nurses do not have a caring attitude and often want to act like doctors and forget that the role of nurse is caring for patient. Indeed, they do not consider the patient and think that care is unnecessary for the patient. ^20^ This finding is in line with the results of our study where some participants pointed out that some care were unnecessary and not important. ^31^

Another theme of this study, living in limbo, also included ethical conflict and the turbulence of existence that participants experienced as missed care. Ethical conflict represented the practice of reprimand, trampling on the patient’s right, discrediting, discriminating in care, being questioned, providing unethical care, feelings of worthlessness and inadequacy, deception, and denial that nursing students had experienced. 

Missing care is an ethical debate that challenges the nurses’ professional and ethical values. Ethical distress is one of the factors that leads to lack of care and mismatch between the patients’ needs and available resources. ^8^ Nurses who experience ethical distress change their circumstances by resigning, reducing working hours, or moving to other wards. Increased medical errors, exacerbated patients’ condition and suffering of their families, and reduced trust and satisfaction in health care systems are the other consequences of missed care. ^8 , 32^ Regarding the nursing students’ denial of missed care, in a similar study it was found that many nurses did not ask the specialist nurses and physiotherapists for the care they had to deliver, but rather assumed that it had been done, because they did not want to know that care was missed. ^25^ Although the reality is that missed care in the clinical setting cannot and should not be denied, when nurses keep silent about it, the opportunity to take corrective action is missed by the staff in next shift. This increases the probability of the recurrence of the same missed care and the risk of negative outcome for patients. ^14^

In the present study, some participants mentioned the role of lack of adequate information and proper training in the occurrence of missed care. Missed nursing care is related to complex factors such as organizational structure, time, healthcare staff, and professional, material, educational and personal characteristics of the nurses. ^6^ Indeed, delays in care, poor evaluation, and inadequate patient management depend on the healthcare staff and educational factors. ^33^ The use of unqualified personnel leads to missed care, which is manifested by delays or failure to provide services. ^20^ The complexity of workload may also put the nurses in a position to make difficult choices about prioritizing care, ^34^ which is consistent with the findings of the present study.

The turbulence of existence represented feeling of regret, mental and emotional struggle, stress and disappointment perceived by nursing students. Many studies indicated that nurses often had concerns about missed patient’s care, emotional pressure, and impaired teamwork, the points that were also observed in the present study. ^6^ Nurses described their experiences as moral distress, role disorder, emotional exhaustion, discomfort, concern, dissatisfaction, and role of stress in the face of failure to provide complete care for their patients. ^35 , 36^ Missed care is one of the key factors in the nurses’ satisfaction with working in clinical setting. ^34^ In a study that evaluated the impact of missed nursing care on nursing staff’s job satisfaction, the results showed that nursing staff who perceived missed nursing care less were more satisfied with their job. ^37^ Another study listed the consequences of missed care, which included disappointment, helplessness, stress, vulnerability, and loneliness, similar to the findings of the present study. ^6^

This study had some strengths and limitations. This research is one of the first studies conducted on this phenomenon among graduate nursing students in Iran. The students had a rich experience of missed care and eagerly shared their experiences. One of the limitations of this study was that it did not include all the experiences of students living in other cities.

## CONCLUSION

Missed care is experienced as unfulfilled care and living in limbo for postgraduate nursing students, which is influenced by organizational and personal factors. The message of this study to policymakers, health care providers, managers, and scholars is that missed nursing care needs to be given greater priority in today’s health care system. Missed care and unfulfilled care is a situation that, if not managed, could put the future of nursing profession at serious risk. It seems that managers can prevent missed nursing care by applying more supervision on nursing care, obtaining feedback on nurses’ performance, reducing the nurses’ workload, creating a culture of teamwork, creating a sense of commitment to work, and enforcing ethical issues among the nurses. 
